# Thermal comfort optimization through bioclimatic design in Mediterranean cities

**DOI:** 10.12688/f1000research.73017.1

**Published:** 2021-10-15

**Authors:** Nermine Hany, Hala Alaa

**Affiliations:** 1 Architectural Engineering and Environmental Design Department, College of Engineering & Technology, AASTMT, Alexandria, Egypt; 2 Post Graduate Student at Architectural Engineering & Environmental Design, College of Engineering & Technology, AASTMT, Alexandria, Egypt

**Keywords:** bioclimatic design, thermal comfort, energy-efficient house, energy performance simulation, Mediterranean architecture, Alexandria

## Abstract

**Background: **Bioclimatic design is an approach based on local climate which improves thermal qualities and indoor comfort. Buildings follow this process to minimize negative effects on the environment. However, this approach is still not suitable in developed countries. This study aims to investigate Mediterranean local bioclimatic strategies’ impact on thermal comfort efficiency in housing, by examining architectural elements and treatments.

**Methods: **We adopted a descriptive, analytical, and comparative methodology, complemented with a software simulation, within a qualitative and quantitative approach. Investigation and methodological tools were based on technical information including plans, elevations, photos, and documentation. The approach consisted of multiple stages: a literature review interpreting the concept of bioclimatic design, as well as thermal comfort variables and common Mediterranean building features. Moreover, the paper showcases three examples of successful Mediterranean passive houses. Furthermore, the paper presents a case- studyhouse in Alex West, Alexandria, designed in the Mediterranean Revival style.

**Results: **The results showed that the most influencing building features on thermal comfort were the low-pitched roofs and the top chimney, which achieved 12.6% and 5% improvement in the summer and 13% and 6.8% in winter, respectively. The pergola and porch elements barely had an effect when placed on the northern façade. However, on the southern façade, a positive contribution in the summer by 1.4% and 3.4% respectively were reported, but a slight negative impact in winter by 0.5% and 2% respectively.

**Conclusions** **:** **We examined the impact of common Mediterranean building features , and compared thermal comfort results between case-study houses. Features focusing on passive design for cooling rather than heating, allowing wind flow for maximized natural ventilation, using ventilated pitched roof spaces, using sun shading elements in the proper facades and angles, help passive thermal regulation. The study proposes recommendations for optimizing thermal comfort in residential buildings in Alexandria, Egypt.

## Introduction

For many years, architecture has been influenced by context and societies. People in different regions had different construction methods depending on their culture, weather, geography, geology…etc. Scientists around the world are developing strategies for reducing a building’s overall energy consumption, in order to minimize negative effects on the environment, while maintaining the desired environmental conditions such as better indoor temperature and thermal comfort. However, this approach is still lacking and not suitable in many regions around the world, especially developing countries such as Jordan Egypt. ^11^ The study aims to investigate the impact of Mediterranean local bioclimatic strategies on thermal comfort efficiency in housing, by examining the architectural elements and treatments that accompanied the Mediterranean building style. The study adopted a descriptive, analytical, and comparative methodology, along with a simulation using DesignBuilder V.6.1 software ^20^ and accompanied with Energyplus core calculations, for the examined case study, within a set of qualitative and quantitative approaches. Investigation and methodological tools were based on technical information including plans, elevations, photos, and documentation.

Previous studies focused on developing a model to be used as a tool by architects, to predict the energy efficiency for buildings in the design phase, as well as describing the architectural strategies employed and analyze the existing trends in bioclimatic architecture. ^1^^,^^3^ The main novelty of this paper is to analyze the impact of Mediterranean local bioclimatic strategies on thermal comfort efficiency in the housing sector, with more specific information was collected on the examined building case study.

The paper consists of multiple sections: first, the concept of bioclimatic design, as well as thermal comfort variables and common Mediterranean local building features in the region are introduced. The rest of this paper is structured as follows. The Methods showcase three examples of successful Mediterranean passive houses. A case study of a house in Alex West, Alexandria, which is designed in the Mediterranean Revival Architectural Style is adopted in the Results. Simulation results are explained in the Discussion depending on the evaluation of scaling parameters. Finally, the last section concludes this work.

## Methods

### Bioclimatic design approach

The concept of bioclimatic architecture is based on taking maximum benefits from the surrounding climate conditions and building placement, to meet indoor thermal comfort needs with minimum energy consumption. ^1^ Bioclimatic architecture is occasionally based on vernacular architecture, and attempts to analyze traditional architecture based on the climate and culture of a place, and to study the architectural and construction solutions. This type of architecture adapts to the local climate without using additional devices that consume energy and leave an ecological footprint. ^2^

To apply bioclimatic architecture, it is necessary to consider the building's location, climate and microclimate; the next step would include the architectural skin, as one of the main elements to consider when striving for comfortable conditions. ^3^

**Figure 1.**
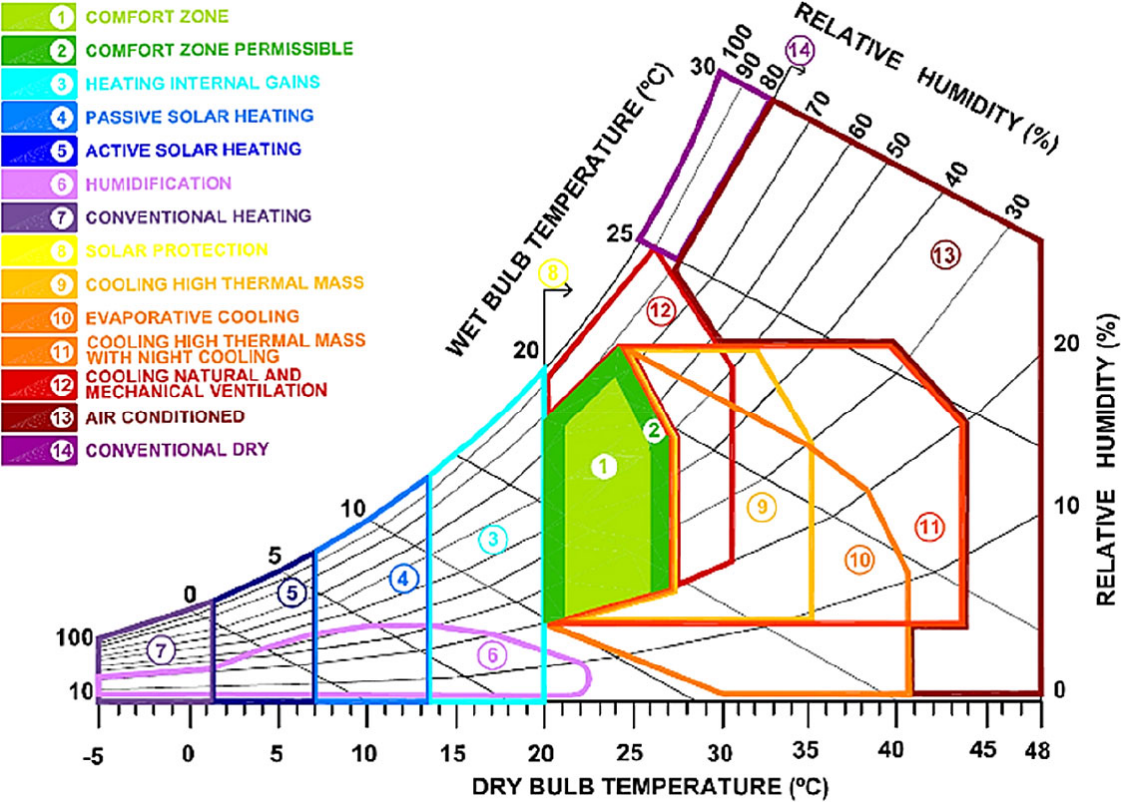
Psychrometric chart adapted from Givoni. ^3^

Different bioclimatic diagrams are used as tools with which to determine comfort levels. ^3^ One of the most widely used tools includes the diagram developed by Baruch Givoni ( Figure 1). The Givoni bioclimatic chart is mainly applied for residential scale construction, and it provides more alternatives in building design to enable thermal comfort, including natural ventilation, evaporative cooling, thermal mass, passive heating, conventional air conditioning or dehumidification. ^4^

*Thermal comfort design variables*

As per ASHRAE 55, thermal comfort is defined as “that condition of mind that expresses satisfaction with the thermal environment and is assessed by subjective evaluation.” The human body is in the constant process of heat exchange with the environment. This heat balance of the human body governs the thermal comfort experience of individuals. As such, many variables affect human thermal comfort. ^5^

Research on thermal comfort focuses on two main factors, namely, the thermal environment factor (including air temperature, relative humidity, air velocity, and mean radiant temperature) and the individual factor (including clothing and metabolic rate). ^6^ Although people could achieve thermal comfort through self-adjustments, such as clothing, activity level, and psychological preference, the thermal environment still plays an essential role in research on thermal comfort. Improving the thermal environment by adjusting architectural forms is one of the most effective methods to achieve thermal comfort. ^7^

**Figure 2.**
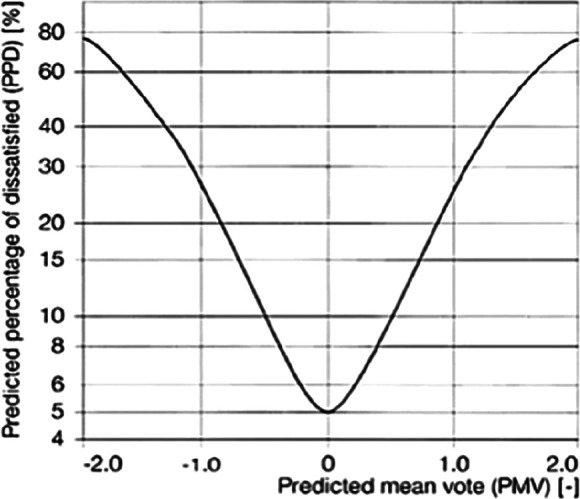
Predicted percentage dissatisfied (PPD) as a function of predicted mean vote (PMV). ^10^

The predicted mean vote (PMV) scale is often used to measure thermal comfort ( Figure 2). The PMV, which was developed by Ole Fanger, is a seven-point scale ranging from −3 to +3 and is the most commonly used thermal comfort index. ^8^ PMV is the mean vote that one would expect to obtain from averaging the thermal sensation votes of a large group of people in a given environment.

The PMV is a complex mathematical expression that involves the individual as well as the four environmental parameters. On the same lines, the predicted percentage dissatisfied (PPD) gives the percentage of people who are dissatisfied with the thermal environment. When PMV is zero, the PPD is of five percent, which means that when the sensational level of cold or hot is zero, five percentage votes are for discomfort. ^9^

### Mediterranean building features

The Mediterranean area is a distinct geographical entity, which has been inhabited since the origins of human history, with particular geomorphologic and climatic features. From Antiquity to the present, cultural trends of East and West intersect and influence each other, and in combination with the natural environment, the climate, the light and the sea, the previous natural elements have defined a very special way of life and consequently, a unique architectural style. ^11^

Mediterranean climate refers to the typical climate of the Mediterranean Basin, and is a particular variety of subtropical climate. ^11^ To state it simply, it usually consists of hot dry summers and mildly cold, wet winters with high daily thermal excursions characterize the Mediterranean climate. Traditional Mediterranean architecture evolved to produce buildings that would be in harmony with the harsh climates of its various regions. In the traditional architecture, the mechanism of indoor thermal regulation was incorporated in the building itself. ^12^

In the Mediterranean region, ventilation and sun protection measures, together with appropriate materials and construction, represent the main issues of bioclimatic efficiency. Ventilation is necessary for comfort and hygiene; even on hot summer days when the outdoors are warmer than the building interior. In traditional buildings, attention was given to ventilation, especially to the pre-treatment of air. Solar shading is important, as well, to control the penetration of the sun in the summer. ^12^

**Figure 3.**
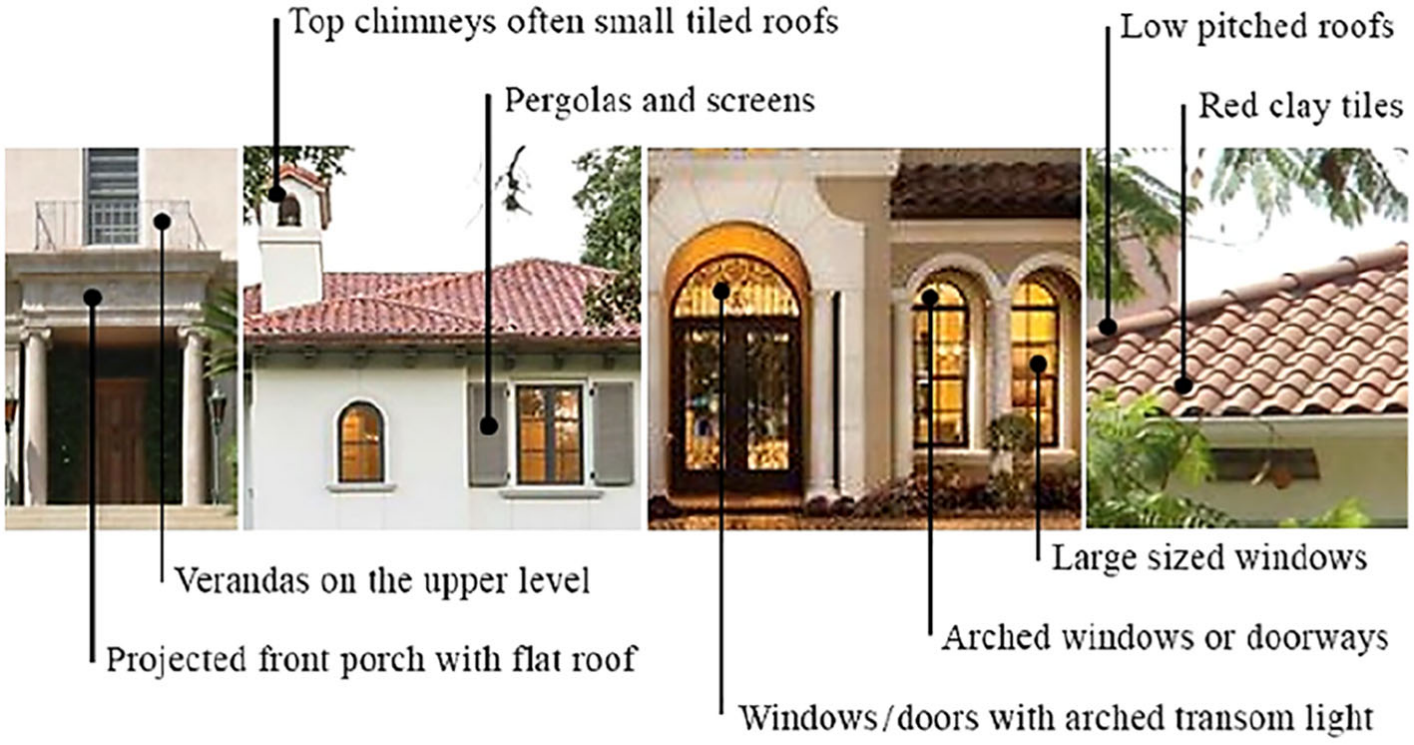
Graphical summary of the common design features in Mediterranean Residential Architecture Style.

The paper summarizes the most common design features in Mediterranean Residential Architecture Style in Table 1, and a graphical summary in Figure 3, based on literature describing Architectural Design Guidelines and its codes; specifically, the Mediterranean Revival Style, as the style incorporated references from various Mediterranean regions such as the Spanish Renaissance, Italian Renaissance, and Arabic Andalusian Architecture.

**Table 1.** Common design features in Mediterranean Residential Architecture Style. ^13^^,^^14^

Element	Design features
Terraces	Verandas on the upper level
	Projected front porch with flat roof
Roofs	Low pitched tile roofs; and occasionally flat
	Red clay tile roofing
External elements	Top chimneys, often small-tiled roofs
	Pergolas and screens
Doors and windows	Large sized windows
	Arched windows or doorways
	Windows and doors with arched transom light
Plan and heights	Open floor plan and high ceilings
	Two-storey buildings

### Examples of good building practices

In this section, the study analyzes three examples of good practice regarding passive and bioclimatic design in Mediterranean cities. The selection criteria of the examples were based on their successful bioclimatic house design approach, their diversity in geographic location, their diversity in passive techniques, and their similarity with the case study. These examples include: Lamaca, Oroklini, Cyprus; Casa Pineda, Barcelona, Spain; and Umbertide, Umbria, Italy.

*Lamaca, Oroklini, Cyprus*

Climate and location

Cyprus has an intense Mediterranean climate with a typical seasonal rhythm concerning temperature, rainfall and weather in general. The predominantly clear blue skies and intense sunshine periods give large seasonal and daily variations between the temperature of the coast and that at the interior of the island, that also is considerably affected by climate change, especially near the coasts. Its average hottest peak reaches 41°C in the summer and drops to an approximate of 5°C in the winter. Relative humidity ranges from 40-60%, and a large daily temperature range is noted with up to 18°C difference between day and night. Thus, Cyprus's climate calls for the need for cooling in the summer, and the large amount of solar radiation during the summer may easily be used for heating in winter. The house is located in the Larnaca District, in the Oroklini village. ^15^ The land is located on a small hill, where neighboring buildings are located at a distance. On the eastern side, there is a road, while to the south, the plot borders a green space, which grants it more privacy.

Building description

**Figure 4.**
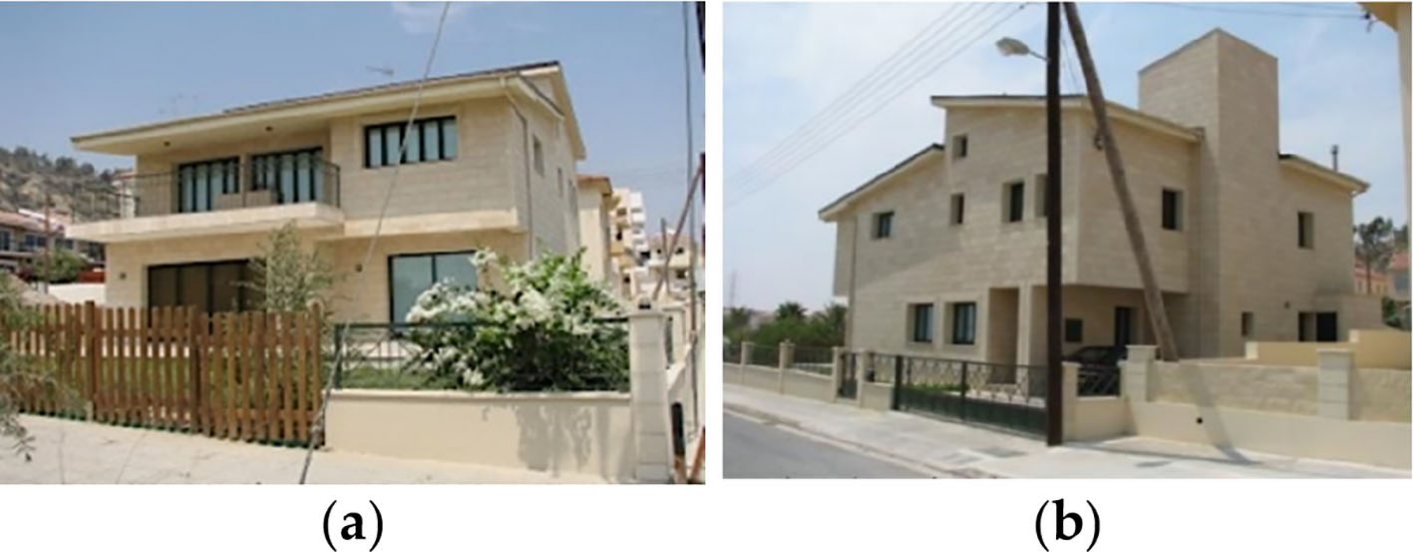
House in Lamaca, Oroklini, Cyprus. ^15^ (a) South façade; (b) North and East façade.

**Figure 5.**
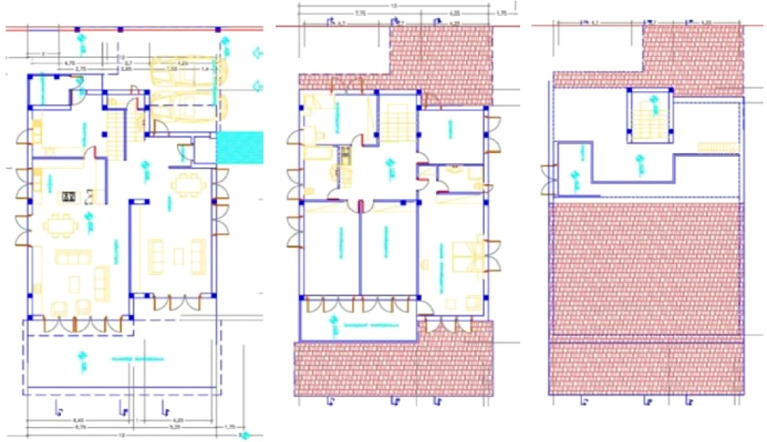
Floor plans of the house in Lamaca, Oroklini, Cyprus. ^15^ (From left to right): Ground floor plan. First floor plan. Mezzanine floor plan.

The building houses a family of four. It consists of three levels ( Figure 4). The ground floor is divided into two individual departments, one of them being accommodated with the entrance, the living room and dining area, while the other department is accommodated with another living room with a dining area and kitchen. The first floor consists of four bedrooms and an office. The mezzanine directly communicates with these areas through an internal staircase ( Figure 5). Situated in a central point on the north with southern clerestory windows which, when opened, give the advantage of direct sunlight gain and contribute to the natural ventilation and stack effect of all spaces on all floors. ^15^

The building frame is reinforced concrete. The external walls are 25cm-thick masonry brick, 5cm-thick thermal insulation, plaster, and stone cladding. The internal walls are 10cm-thick masonry brick wall, plaster, and paint. The roof is inclined reinforced concrete, with 10cm-thick thermal insulation, water barrier, and ceramic roofing tiles. The windows are aluminium profile, double glazed, have low emissivity, and argon filled. Also, the floors are composed of ceramic tiles. ^15^

Bioclimatic Approach

**Figure 6.**
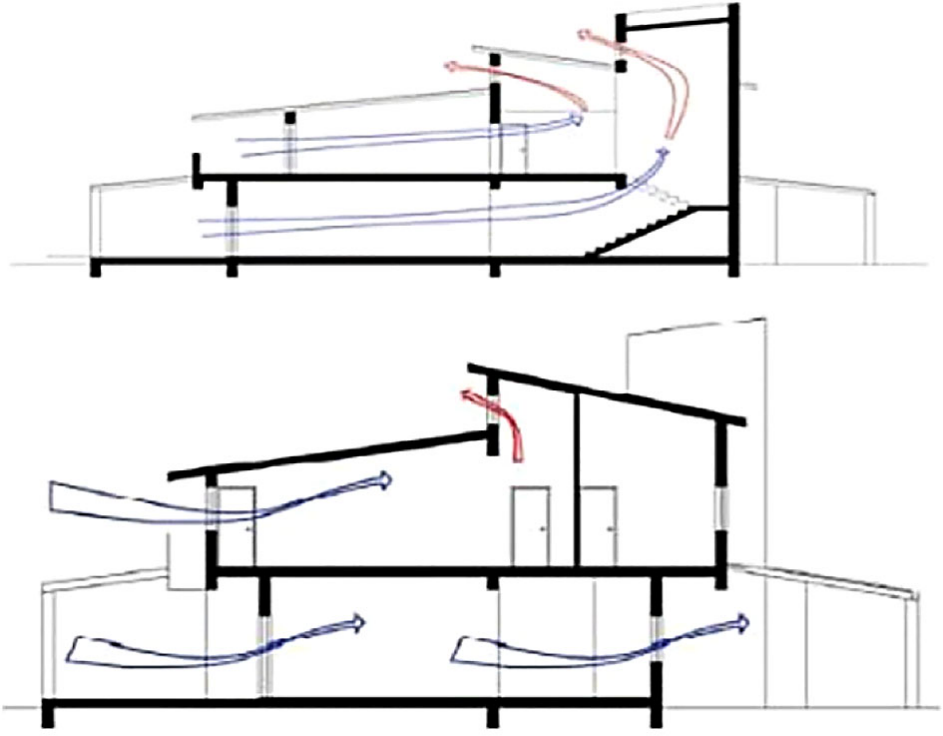
South-north cross sections showing the natural ventilation. ^15^

The bioclimatic approach is applied in; orientation as most spaces are South-oriented, thermal mass in floors, walls, and staircase; passive solar heating as direct gain (glass openings and clerestory windows). Solar control is achieved with external shading devices with regards to East and West. Moreover, the shading of openings is achieved with the use of overhangs, with the extension of the floors on the southern and northern sections and extension of the roof and the balcony on the southern section. There is also anticipation at the time of writing to plant trees around the building shell, but this has not been developed to a satisfactory level yet. The natural ventilation relies on night ventilation (in the summer nights all openings can be opened manually, except the clerestory windows which are electrical), cross ventilation (provisions had been made so that most spaces have openings on two sides), and stack effect ( Figure 6) (At the top point of the staircase as well as in the mezzanine area, clerestory windows have been placed and are opened during the summer months). ^15^

*Casa Pineda, Barcelona, Spain*

Climate and location

The Mediterranean coast of Spain is in the warm climate zone, where average temperatures in July and August are about 26°C. Recent results indicate that summer temperatures may be 3°C to 4°C lower. Winters are moderately cold and humid, so active heating is still necessary for residential buildings. The weather in Palau Plegamans (province of Barcelona) is similar to Barcelona, but the daily temperature oscillation in the summer is more pronounced, with typical temperatures of 14°C in the early morning hours. Located at 41° North, high solar radiation is available during the winter months. ^16^

Building description

**Figure 7.**
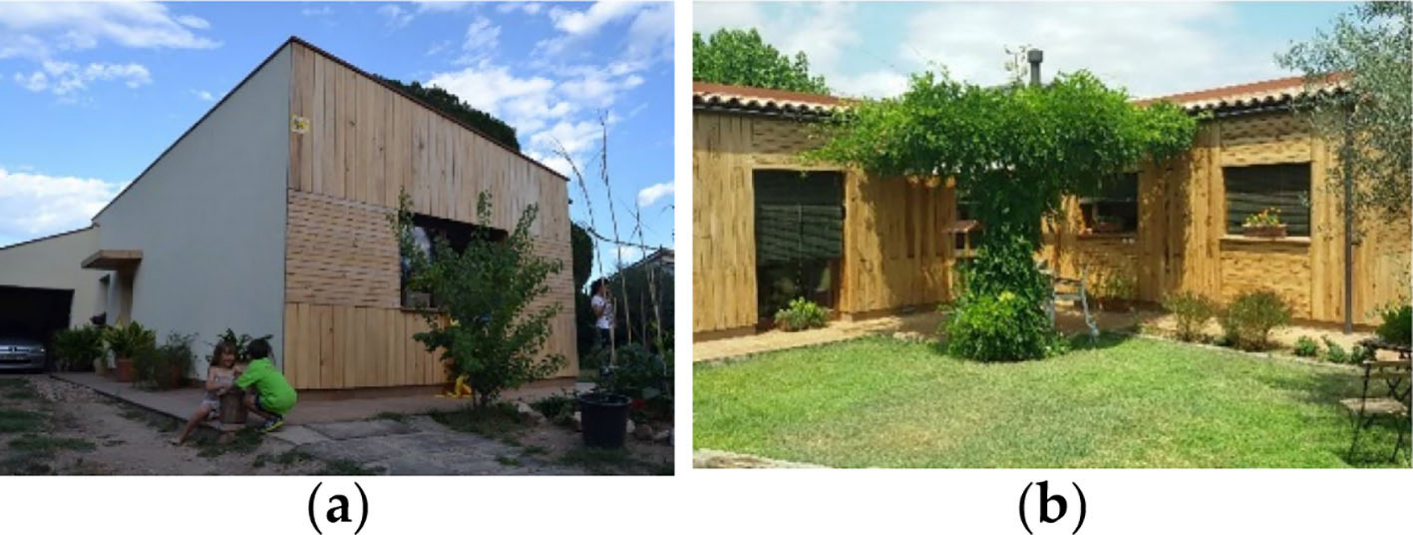
House in Casa Pineda, Barcelona, Spain. ^16^ (a) The southern façade; (b) The eastern façade showing L-shaped structure and open garden.

The detached family house “Casa Pineda” in Palau Plegamans has one story and no basement floor. The user of the building is a two-person family ( Figure 7). It is a lightweight building with a wooden beam structure; the roof is covered with rear-ventilated roof tiles. In Catalonia, it is common to combine passive houses with wood structure, which is not more expensive than the ‘traditional’ construction system, based on burnt bricks. ^16^ The building is located in a suburban district. The L-shaped building is oriented towards the south, defining a homely open space in the garden.

Bioclimatic approach

**Figure 8.**
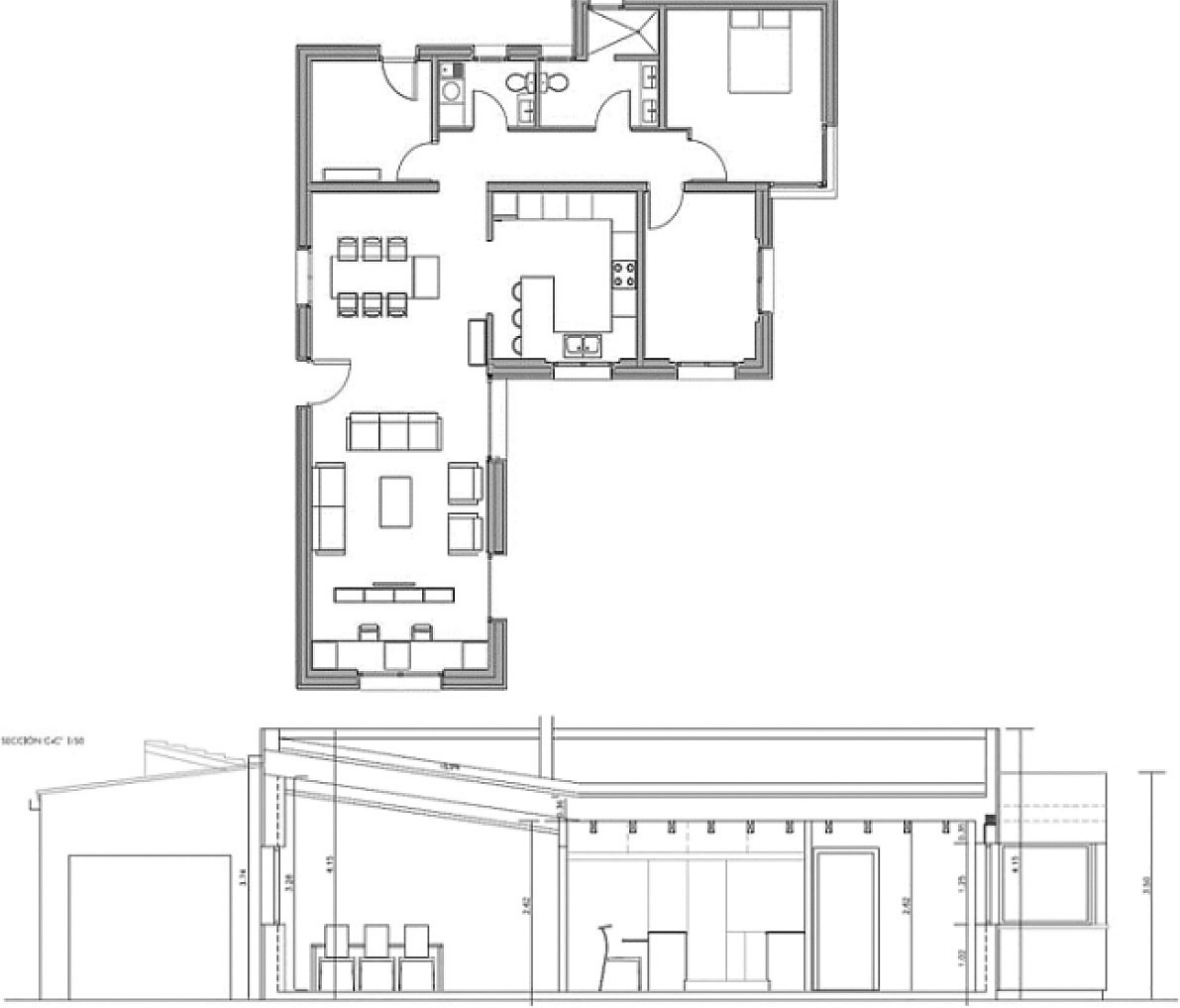
Floor plan and cross section of the house “Casa Pineda”, Barcelona, Spain. ^16^

The building is a lightweight construction with low thermal inertia and there is no active cooling system. An EIFS with EPS insulation was applied to the exterior walls on the northern and eastern side; the other façades are finished with a wooden ventilated structure. The main openings are towards the South (50% of all windows) ( Figure 8), so in winter, the solar gains due to windows are about 40% higher than the transmission losses of the windows. Windows were installed on the inner side of the walls. This solution has a high thermal bridge effect. Summer comfort is achieved by appropriate user behavior: during daytime, closing exterior blinds which reduces mechanical ventilation; during night times, tilting windows and wide opening in the early morning hours. All windows have efficient sun shading blinds. ^16^

*Umbertide, Umbria, Italy*

Climate and location

The project is a development of bioclimatic housing for a housing development in Umbertide, Umbria, in Italy. Umbertide lies in a Mediterranean temperate climate with rainy winters and hot summers. The local climate creates a significant requirement for energy for heating and cooling. During the summer months, light breezes (under five to 10 metres per second) from the South, East or West enter the city from the surrounding hills and are caught by the streets that separate the buildings, providing them with natural air conditioning. During the remaining seasonal periods, the prevalent direction of the wind is North-West. The most comfortable months are in the spring (May) and in autumn (September and October); the central months (from June to August) are hot and humid with light breezes. ^17^

Building description

**Figure 9.**
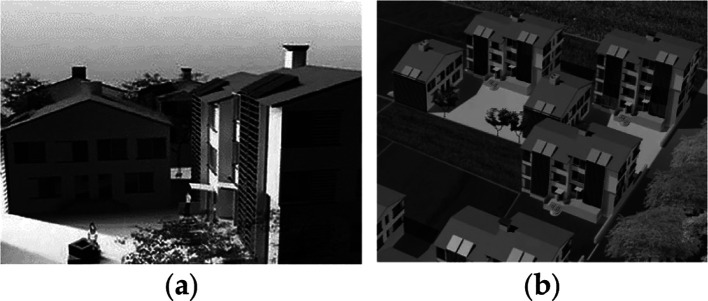
House development in Umbertide, Umbria, Italy. ^17^ (a) Building typology showing the shading screens on the external façade; (b) The housing courtyard.

The alternation of the form and function of this external space is organized according to the position of the site, in correspondence with the main pedestrian wind axis or with the secondary edible garden lines. The apartment buildings range from two to five floors, depending upon different typologies ( Figure 9), and each floor is composed of four rooms (bedrooms, kitchen and living room) and two bathrooms. The construction system is as follows; a structural system for row houses and detached houses consisting of load-bearing walls of traditional brick masonry; apartments are made from reinforced concrete frames with traditional brick cladding; external walls comprising ventilated cavities, with traditional brick masonry; roofs comprising a concrete structural system, sealer, air space and traditional roof tiles. ^17^

Bioclimatic approach

**Figure 10.**
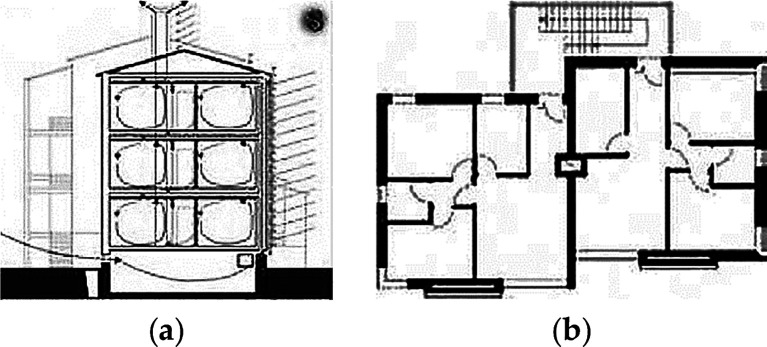
The apartment house in Umbertide, Umbria, Italy. (a) Cross section showing predicted ventilation system; (b) Typical Floor plan.

At the building level, the use of the stack effect (integrated with a convective loop system and with a chimney) to realize a system of cooling and ventilation resulted in a good microclimate and adequate indoor conditions during summertime. The development of this system has been progressive and has been tested and improved by continuous fluent simulations. Incoming air is transported through the building by different chimneys, either for admission or for expulsion functions, which work by stack effect ( Figure 10). In the admission chimney, during winter, the air flows into the glazed section, becomes warmer as a result of the passive greenhouse effect and is then distributed to each room. In summer, the air flows into an insulated section and provides cooling due to its speed and continuous circulation; moreover, shading systems at the windows and at the glazed chimney prevent overheating. Part of the south façade is completely glazed in order to increase direct heat gain. Sun protection in summer is provided through selective shading, which allows winter direct heat gain from the sun, but screens out summer sun. ^17^

*Examples of mediterranean muilding features*

The study analyzed three examples of passive housing in Mediterranean cities, which have managed to achieve sustainable design and improved building performance, along with maintaining the Mediterranean architecture style. The study concluded that the most common Mediterranean local building features utilized in passive building design based on these examples are low-pitched roofs and large sized windows, followed by a projected front porch, red clay roofs, pergolas and screens, top chimneys, open floor plan and high ceiling, as well as two-story buildings. Table 2 summarizes the achieved features in the examples, to present a framework for the selection of the examined building features in section 4.

**Table 2.** Mediterranean design features achieved in the analyzed examples.

Design features	Lamaca	Casa pineda	Umbertide
Verandas	✓		
Projected front porch		✓	✓
Low-pitched roofs	✓	✓	✓
Red clay tile roofs	✓	✓	
Top chimneys	✓		✓
Pergolas and screens		✓	✓
Large-sized indows	✓	✓	✓
Arched windows or doorways			
Windows and doors with arched transom light			
Open floor plan and high ceilings	✓	✓	
Two-story buildings	✓	✓	

## Results

The paper investigates the impact of local Mediterranean building features on the thermal comfort of a house in Alexandria. First, it discusses the climatic characteristics of the location, and analyzes the bioclimatic chart. Furthermore, it examines the performance of the house of the case study, with and without the tested building features.

### Bioclimatic chart analysis

The approach considered here included performing a preliminary analysis of the bioclimatic chart to formulate the general design strategies that are most adapted to the climate of Alexandria. A didactical software named Climate Consultant v. 6.0 ^21^ was used for this purpose. This tool, based on the Milne and Givoni bioclimatic chart, ^18^ plots the climatic data (i.e., dry bulb temperature and relative humidity data) on the psychrometric graph. The distribution of the hourly climatic data on the psychrometric graph provides the relative potential of the application of each bioclimatic design strategy (passive and active) to improve indoor thermal comfort.

Among the different thermal comfort models proposed by this tool, the ASHRAE Handbook of Fundamentals 2005 Comfort Model was chosen. For people dressed in normal winter clothes (relatively thicker with warmer materials), at effective temperatures of 20°C to 23.3°C (measured at 50% relative humidity), the temperature decreases slightly as humidity rises. The upper humidity limit was a 17.8°C wet bulb and a lower dew point of 2.2°C. If people are dressed in light weight summer clothes, then this comfort zone shifts 2.8°C warmer.

**Figure 11.**
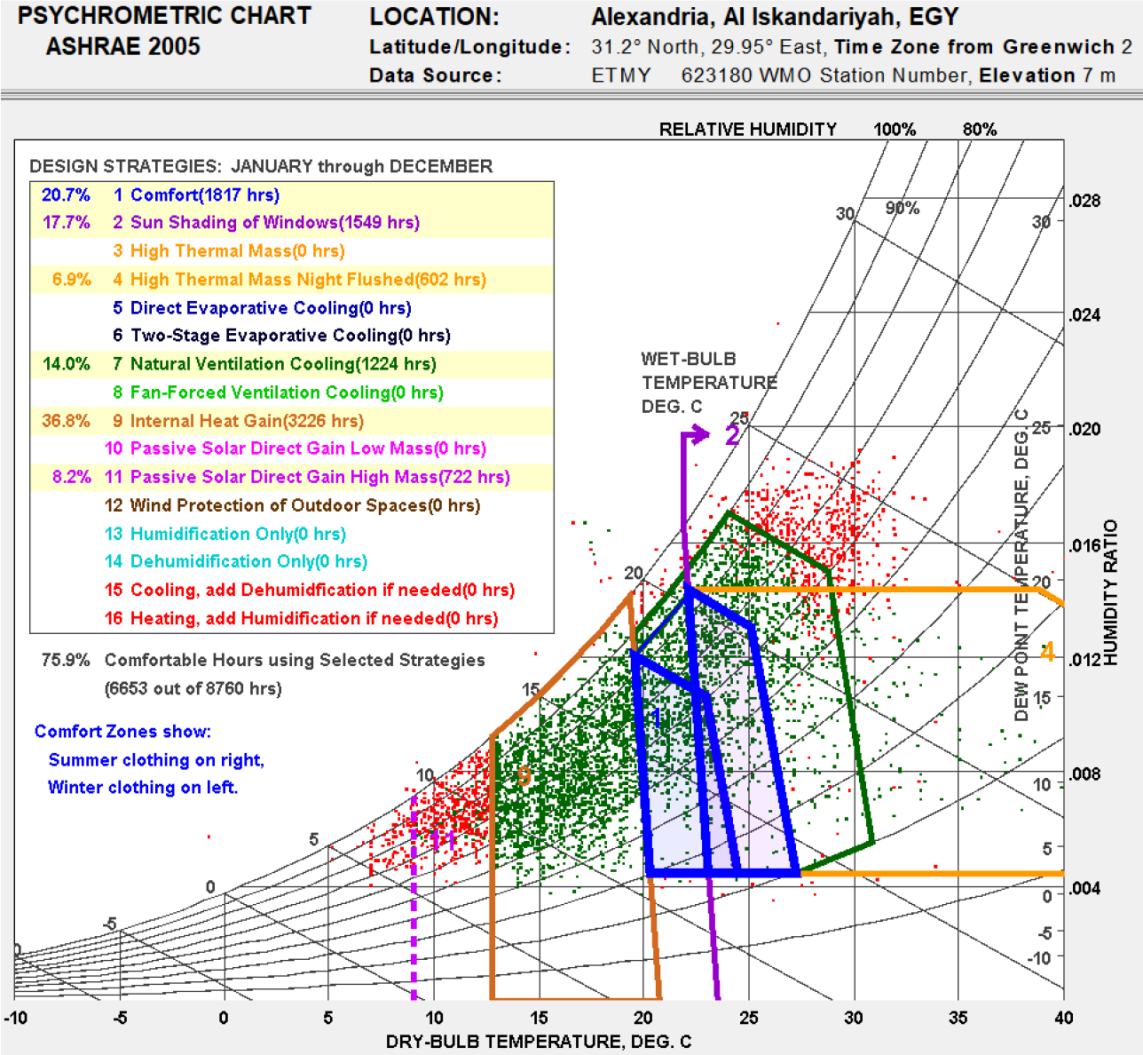
Bioclimatic chart for Alexandria generated using the Climate Consultant tool using recommended passive design strategies. Green represents ‘comfortable’ hours by 76%. Red represents ‘not comfortable’ hours by 24%.

The bioclimatic chart of Alexandria city, obtained using Climate Consultant, is presented in Figure 11, displaying only the passive design strategies. It is noticeable that the percentage of the comfort zone did not exceed 20.7% of the year. The exploitation of the internal heat gains should extend the number of thermal hours of about 36.8% primarily during the cold season. On the second level, sun shading from windows could represent an enhancement of about 17.70%, in particular in the hot season. The natural ventilation cooling contributed to 14%, as well as high thermal mass night flushing by 6.9%. The passive solar gain contributed to about 8.2% when combined with high thermal mass, allowing the comfort level to reach 76%. However, the previous outcomes only provided general recommendations for design enhancement, given that it was based solely on the analysis of the climatic data and did not take into account the specificities of each building case. Thus, design parameters would be further specified for the given case study in the following analysis.

### Case study building description

**Figure 12.**
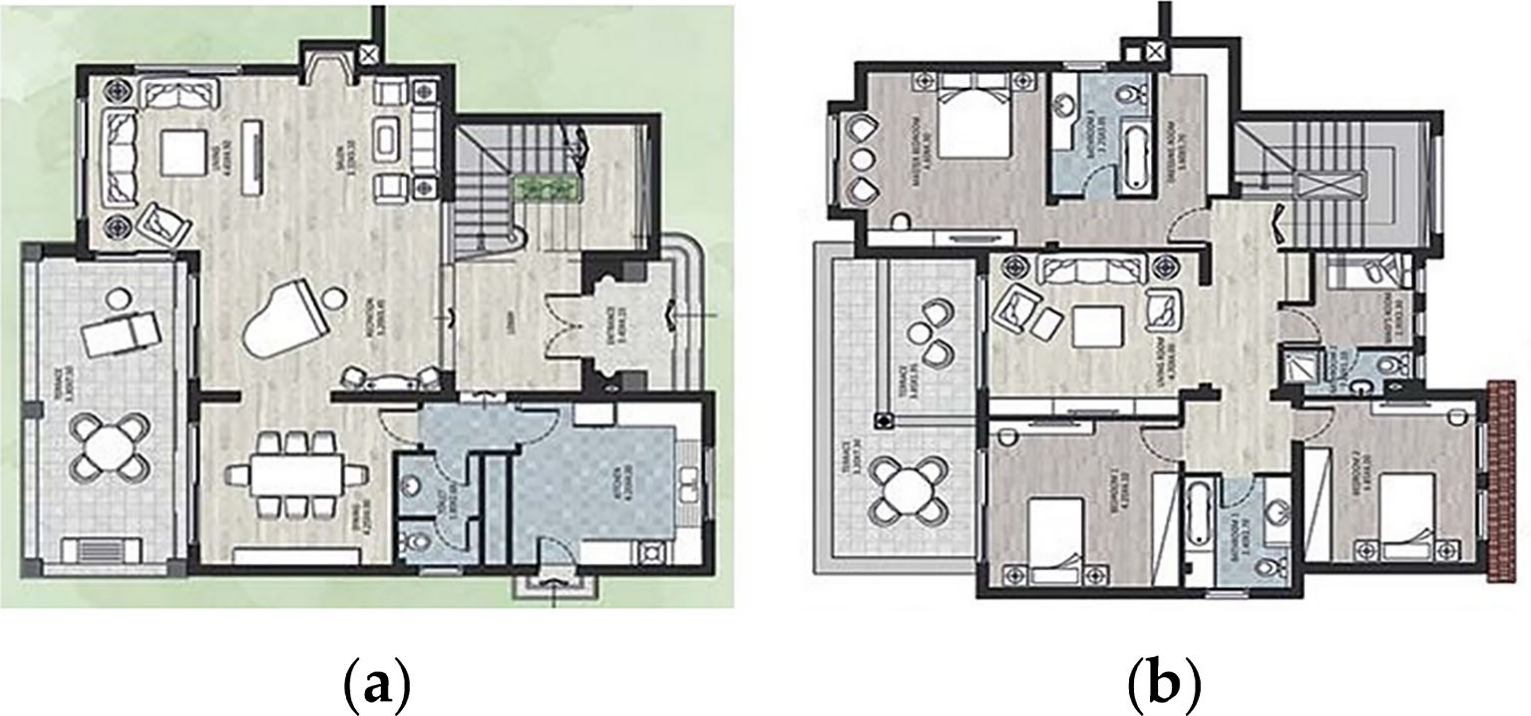
Floors plans of the house in Alex West, Alexandria, Egypt. ^19^ (a) Ground floor plan; (b) First floor plan.

**Figure 13.**
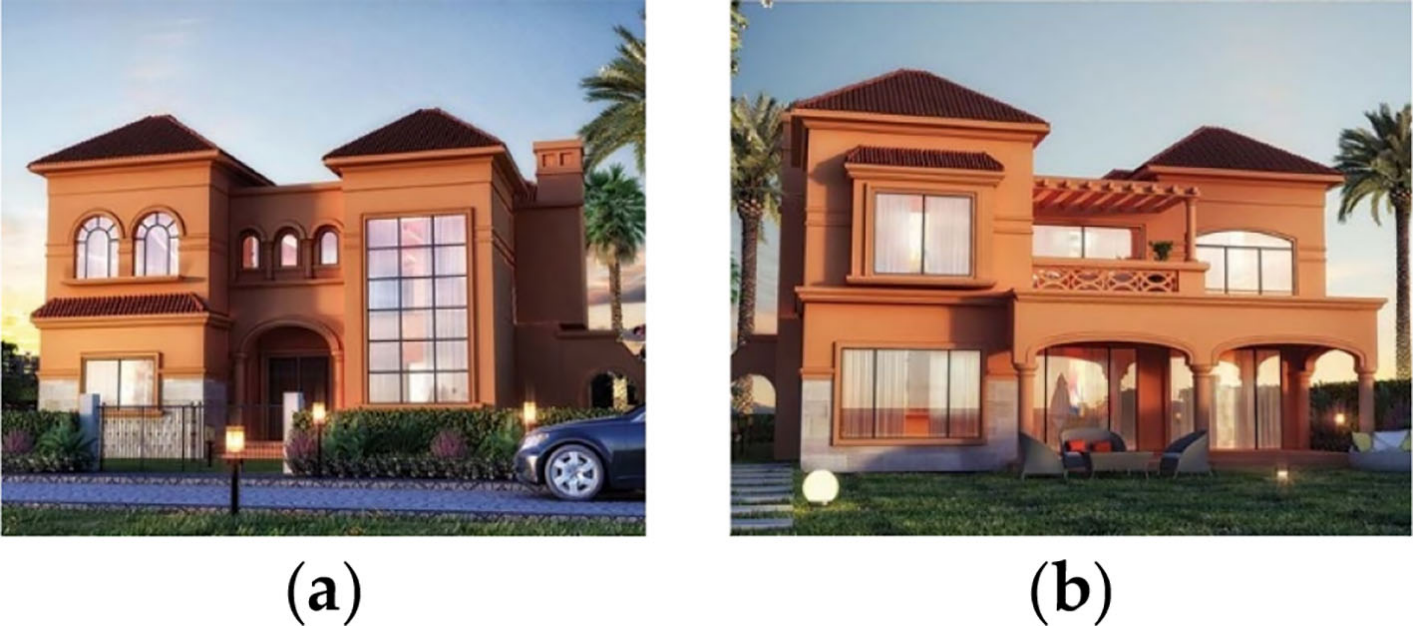
House in Alex West, Alexandria, Egypt. ^19^ (a) Southern façade shot; (b) Northern façade shot.

For the purpose of this paper, a case study of a detached family house was considered. The house is located in the Alex West compound, in Alexandria. The chosen prototype was V4, which is located in the St. Catherine zone. It consists of two floors, four bedrooms, a living room, a dining room, a kitchen, four bathrooms, and is occupied by five people ( Figure 12). The total built-up area is 312 m ^2^ with a 3-metre height. The building design follows the Mediterranean Revival architectural style ( Figure 13).

**Figure 14.**
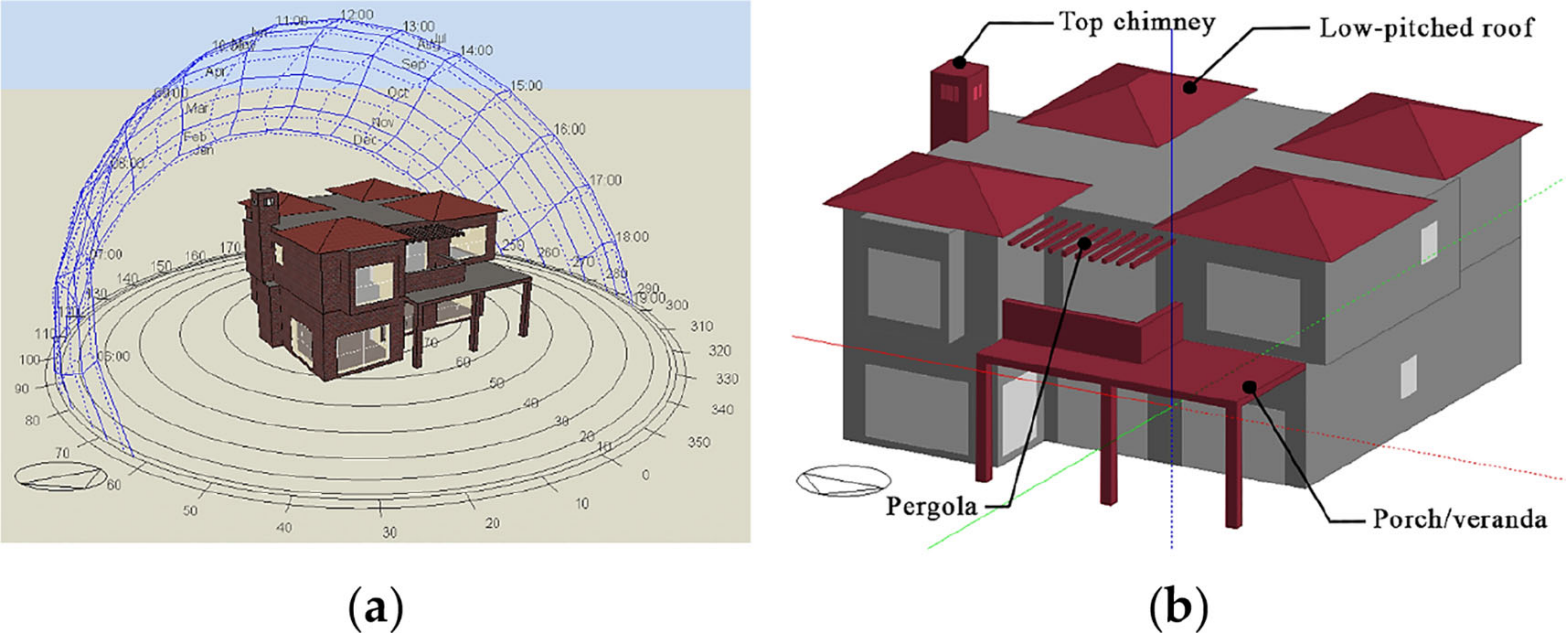
Three-dimensional model of the house in Alex West, Alexandria, Egypt, constructed with DesignBuilder. (a) DesignBuilder view; (b) Mediterranean building features subject to investigation.

The software used for analysis was DesignBuilder v. 6.1 ^20^ ( Figure 14). The building was constructed and analyzed, and the boundary conditions were imported as output data of EnergyPlus dynamic thermal simulation. ^22^ Furthermore, the paper set specific local building features for investigation, based on the literature review, as well as the most common features drawn from the analysis of the successful passive building examples. These features include the low-pitched roof, the pergola, the projected porch/veranda, and the top chimney ( Figure 14).

The dynamic simulation tool was used to estimate the occupant thermal comfort PMV levels, as it incorporates an AUTOCAD interface for the EnergyPlus core calculations. ^22^ Site data is based on WMO meteorological measurements for the egyptian typical meteorological year (ETMY) reported by the Alexandria station number 623180. The project file settings specified in the software are summarized in Table 3 including heating, ventilation and air conditioning (HVAC), environment, and activity settings. As the research focuses on passive design techniques, the system was set to natural ventilation only, avoiding any active heating, cooling, mechanical cooling, or ventilation control to take effect. Furthermore, the materials specified in the project are showed in Table 4. The top chimney feature is made of the material type ‘solid brick 125 mm, uninsulated’, the low-pitched roof feature of ‘clay tiles 25 mm on concrete 150 mm, reinforced with 2% steel’, the porch/veranda feature of ‘solid brick 250 mm, uninsulated’, and finally the pergola feature is made of the material type ‘2”×6” timber wooden stringers’.

**Table 3.** Case study project file settings assigned in DesignBuilder. HVAC: heating, ventilation and air conditioning.

DesignBuilder project file settings	
HVAC template	Natural ventilation –No heating/cooling
Mechanical ventilation	Checked off
Humidity control	Checked off
Natural ventilation operation schedule	Dwell_DomCommonAreas_Occ *
Indoor ventilation setpoint temperature	20 degree celsius
Activity template	Residential spaces
Occupancy density	0.16 people/m ^2^

* An operation schedule for ventilation assigned in DesignBuilder and set for residential spaces common areas.

**Table 4.** Case study project construction materials assigned in DesignBuilder.

Project construction materials	
External walls	Solid brick wall 250 mm, uninsulated
Internal partitions	Solid brick wall 125 mm, uninsulated
Flat roofs	Concrete 200 mm, reinforced with 2% steel
Pitched roofs	Clay tiles 25 mm on concrete 150 mm, reinforced with 2% steel
Openings	Single glazing reflective 6 mm clear glass
Pergola	2” × 6” timber wooden stringers

### Simulation results

**Figure 15.**
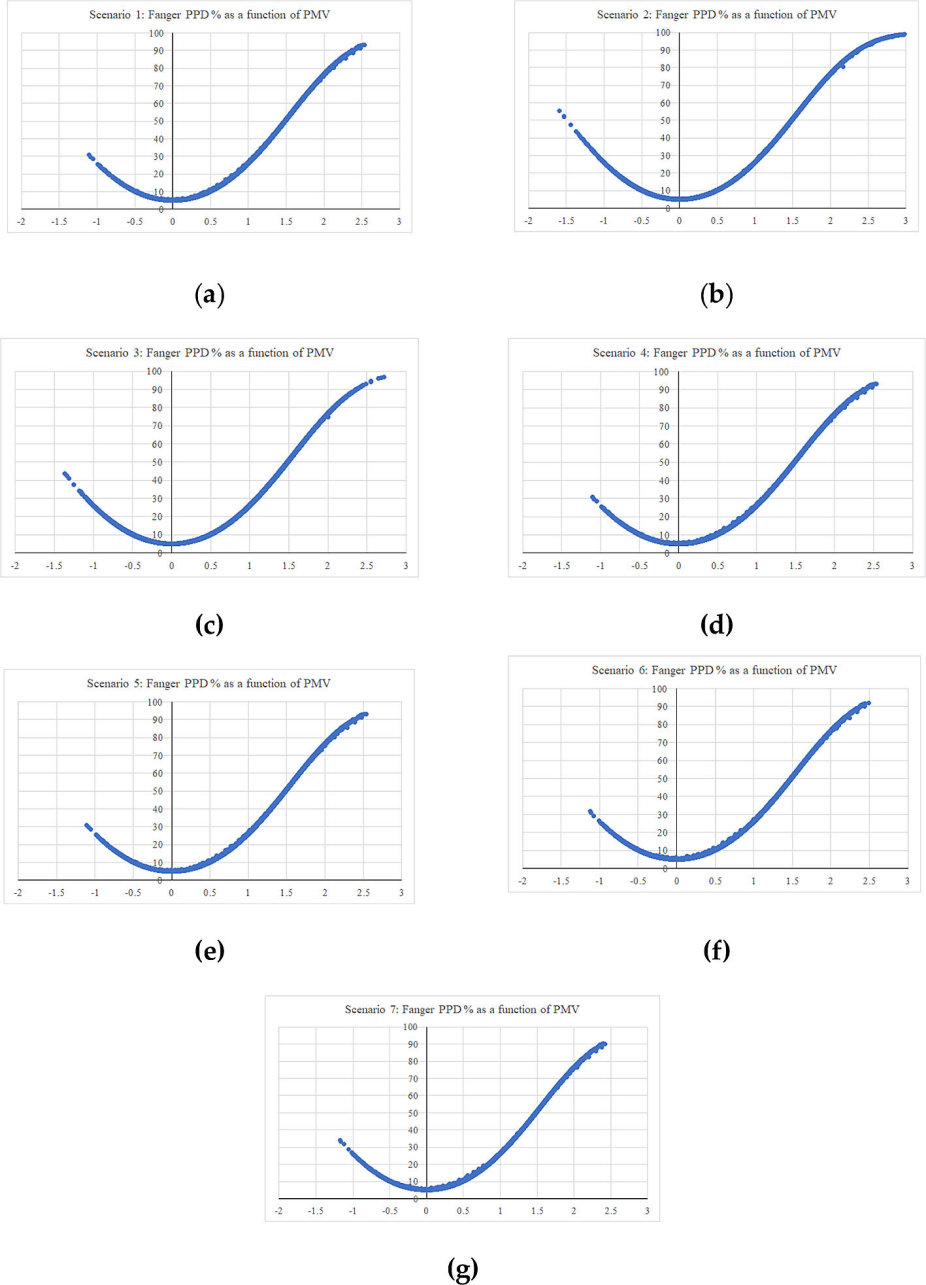
Fanger PPD as a function of PMV (a) hourly simulation result for scenario 1 – the base case; (b) hourly simulation result for scenario 2; (c) hourly simulation result for scenario 3; (d) hourly simulation result for scenario 4; (e) hourly simulation result for scenario 5; (f) hourly simulation result for scenario 6; (g) hourly simulation result for scenario 7.

The simulation period was assigned for the entire year from 1 January to 31 December, within hourly output intervals. Seven simulations were conducted; one for the base case, while the other six were modified scenarios for the same building aimed at testing the specified Mediterranean building features. Table 5 summarizes the simulation scenarios. Scenarios 1-5 (S1-5) included removing the examined building features (low-pitched roofs, top chimney, pergola, and porch/veranda) which already existed in the building. However, S4 and S5 results had barely shifted from the base case result, due to the position of the pergola and porch on the northern façade. Therefore, S6 and S7 were tested as well, which consisted in examining the cases of moving the same pergola and porch elements to the southern façade. Figure 15 shows the graphical and numerical representation of the resulting hourly simulation outputs for Fanger PPD percentage as a function of PMV, in the whole thermal zones of the examined building.

**Table 5.** Case study for the seven simulation scenarios. w/o = without.

Scenario	Description
S1	base case
S2	w/o low pitched roofs
S3	w/o top chimney
S4	w/o pergola
S5	w/o porch/veranda
S6	with pergola (south)
S7	with porch/veranda (south)

## Discussion

Thermal comfort was examined for the previous building scenarios, using Fanger PPD and PMV values, to indicate the percentage of dissatisfaction of users in the building for these conditions. Table 6 demonstrates the PMV scaling parameters ranging from −3 to 3; a PMV of −3 represents the cold perception, while a PMV of 3 represents the hot perception. The simulation we carried out showed that the examined building features have made some considerable alleviation in both summer and winter.

**Table 6.** PMV scaling parameters.

Perception	Predicted mean vote (PMV)
Cold	−3
Cool	−2
Acceptably cool	−1
Neutral (comfortable)	0
Acceptably warm	1
Warm	2
Hot	3

The first scenario examined the thermal comfort for the base case, which included all the villa’s existing building features. The PMV reached a maximum output value of 2.51 on the hot scale, and a value of −1.10 on the cold scale. On the other hand, the second scenario, which examined thermal comfort in the case the low-pitched roofs element was removed, and replacing them with flat roofs, showed the greatest alleviation among all the scenarios on both the hot and cold scales. The result reported a maximum output value of 3 on the hot scale, which represents a 12.6% increase, and a value of −1.58 on the cold scale, which represents a 13% increase from the base case. The third scenario, which examined thermal comfort in the case the top chimney element was removed, had reported a maximum PMV output value of 2.71 on the hot scale with a 5% increase, and a value of −1.63 on the cold scale with a 6.8% increase. This scenario was the second greatest alleviation among the other ones.

**Figure 16.**
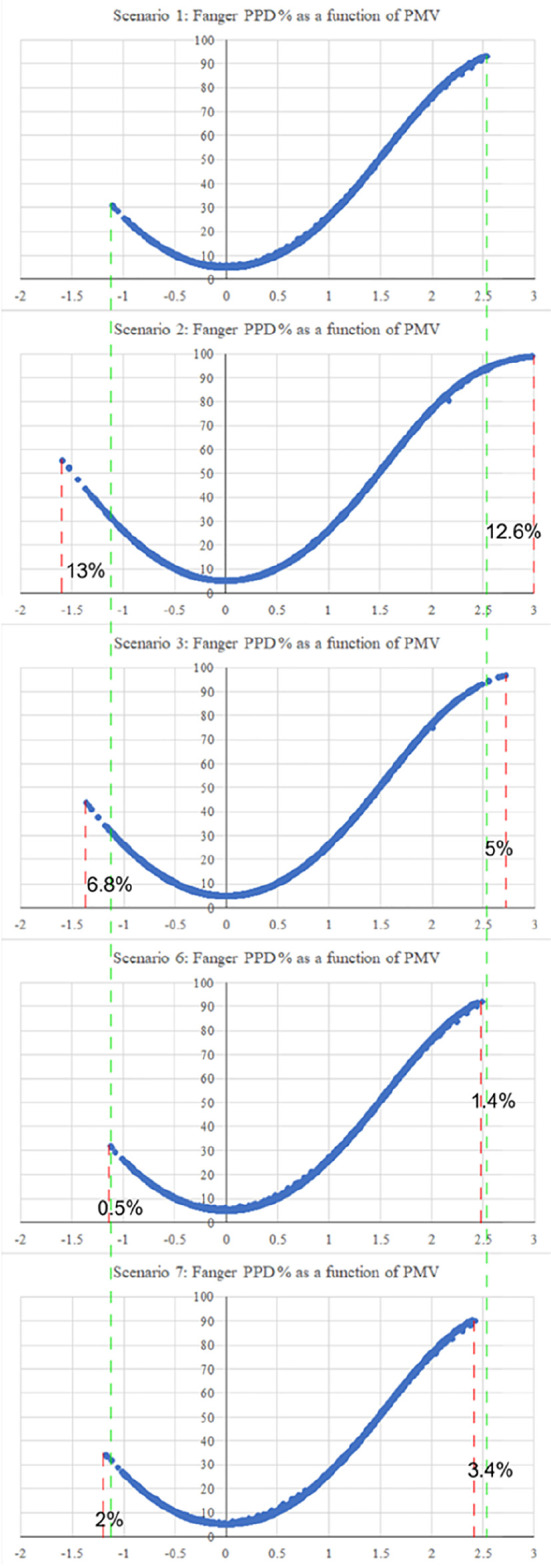
Alleviation and aggravation of the results for the various scenarios compared to the base case. The green dashed lines represent the maximum PMV values of the base case on both the hot and cold scale. The red dashed lines represent the shift of results in the other scenarios.

Scenarios 4 and 5 barely showed any alleviation due to the northern façade position of the pergola and porch elements, which barely caused any auxiliary shading effect nor impacted thermal comfort. Therefore, this research examined the impact of the same pergola and porch elements in the southern façade respectively. Both techniques caused a slight alleviation in the summer, but also a slight aggravation in the winter. Scenario 6 included adding the pergola element in the southern façade, and results reported a maximum PMV output value of 2.49 on the hot scale with a 1.4% reduction from the base case, and a value of −1.11 on the cold scale with a 0.5% increase from the base case. Scenario 7, which examined moving the porch element to the southern façade, had reported a maximum PMV output value of 2.39 on the hot scale with a 3.4% reduction from the base case, and a value of −1.16 on the cold scale with a 2% increase from the base case. Figure 16 shows the graphical shift of results from the base case for the various scenarios.

**Figure 17.**
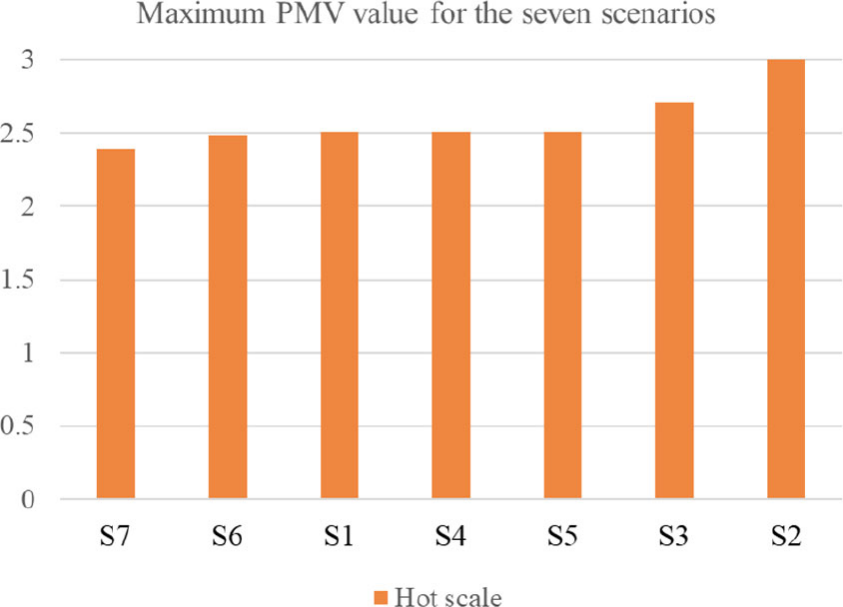
Resulted Fanger PMV value for the seven scenarios ordered from best to least convenient bioclimatic design on the hot scale.

**Figure 18.**
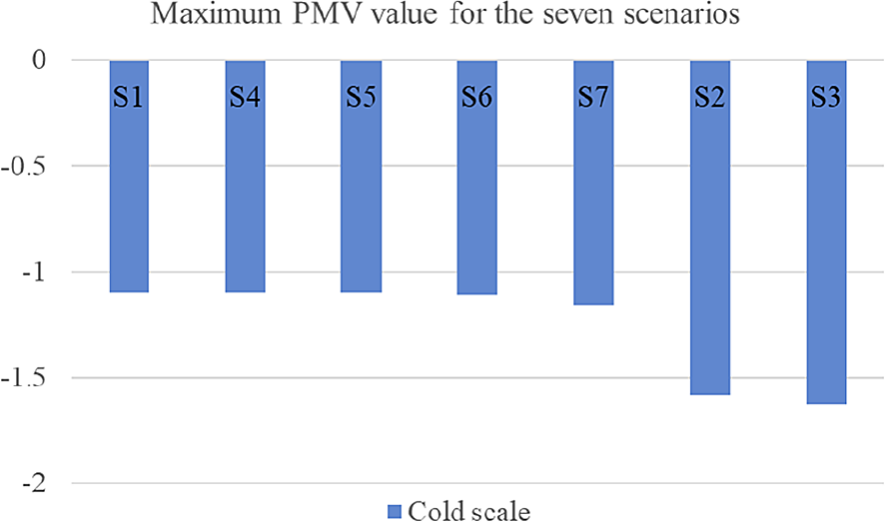
Resulted Fanger PMV value for the seven scenarios ordered from best to least convenient bioclimatic design on the cold scale.

Table 7 compares the maximum resulting PMV for the seven tested scenarios for both the hot and cold scales. Moreover, Figures 17 and 18 illustrate the maximum PMV from best to least convenient bioclimatic design, for hot and cold scales respectively.

**Table 7.** The maximum Fanger PMV for the seven scenarios on both hot and cold scales resulting from case study simulation.

Scenarios	Max PMV on hot scale	Max PMV on cold scale
S1	2.51	−1.1
S2	3	−1.58
S3	2.71	−1.63
S4	2.51	−1.1
S5	2.51	−1.1
S6	2.49	−1.11
S7	2.39	−1.16

**Figure 19.**
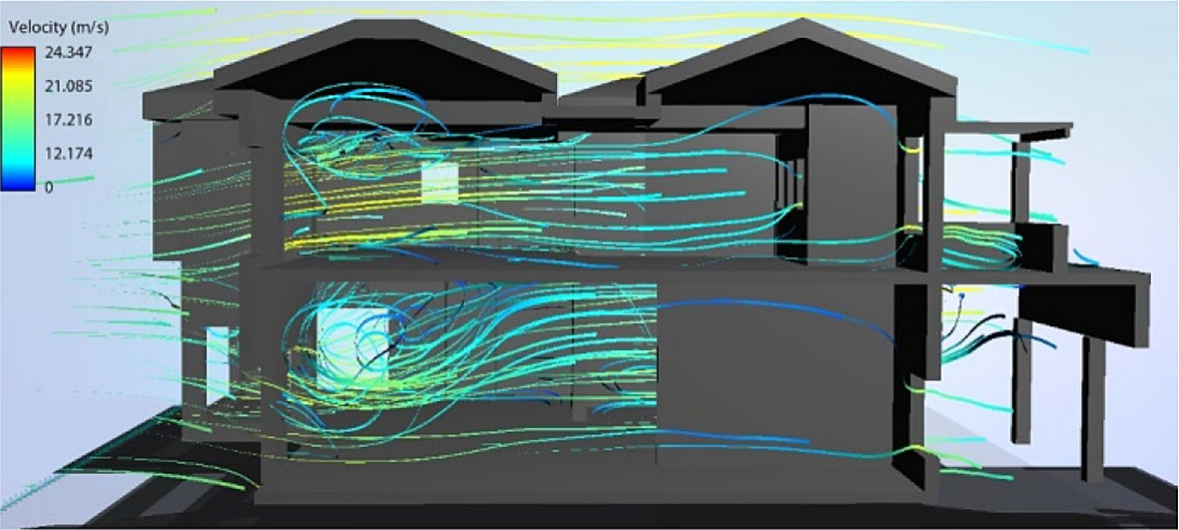
North-South cross section of the case study building showing the natural ventilation flow for the best bioclimatic design alternative (S7).

**Figure 20.**
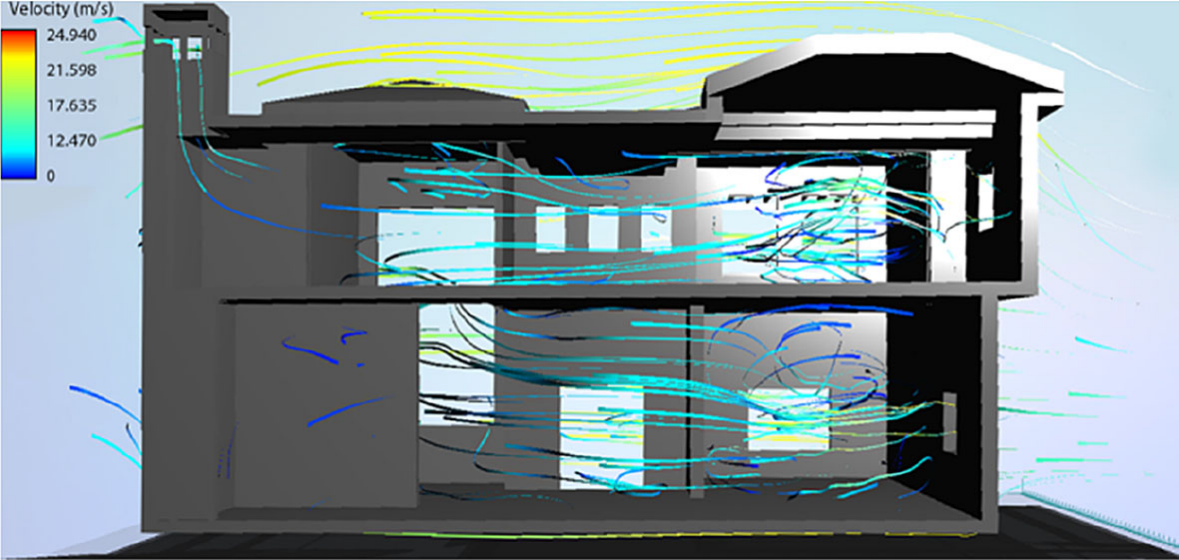
West-East cross section of the case study building showing the natural ventilation flow for the best bioclimatic design alternative (S7).

Overall, for a yearly thermal comfort outcome, the best bioclimatic design alternative was determined to be S7. The Flow Design software was used to generate the natural ventilation flow for the building’s cross sections, showing the impact of the tested Mediterranean features in the best design scenario alternative concluded from the simulation ( Figures 19 and 20).

## Conclusion

The bioclimatic design concept aims to improve building thermal performance with a perfect adaptation to the local climate specificities. The PMV scale, which was developed by Ole Fanger, is a seven-point scale ranging from −3 to +3 and is the most commonly used thermal comfort index. Moreover, this paper summarizes the most common design features in the Mediterranean Residential Architecture Style. The analytical section included analyzing three examples from Lamaca, Oroklini, Cyprus; Casa Pineda, Barcelona, Spain; and Umbertide, Umbria, Italy. Consequently, the paper concluded the most common design features from the literature and examples examined in the case study, were low-pitched roofs, top chimney, pergola, and porch/veranda.

For the paper’s case study, a bioclimatic chart was analyzed to highlight the passive design strategies which are the most adapted to the climate in Alexandria, Egypt. The bioclimatic chart showed that the internal heat gain, sun shading of windows, and natural ventilation were the most optimal passive design strategies to increase thermal comfort range in the city of Alexandria. The building adopted for case study analysis was a single family detached house in Alex West, Alexandria, Egypt; it was designed in the Revival Mediterranean architectural style, consisted of two floors, and was occupied by five people. Simulations were carried out on the building’s thermal zones following seven different scenarios, to examine the impact of removing each building feature on thermal comfort.

The overall building thermal comfort in all scenarios yielded better results in the winter than in the summer. The results showed that the most influencial building features on thermal comfort of the building were the low-pitched roofs and top chimney elements, which achieved 12.6% and 5% improvement in the summer and 13% and 6.8% in winter, respectively. The pergola and porch elements barely had an effect when placed on the northern façade. However, replacing their position to the southern façade resulted in a positive contribution in the summer by 1.4% and 3.4% respectively, but also a slight negative impact in the winter by 0.5% and 2% respectively.

It was clear that the such pre-existing local building features had an overall positive impact on the thermal comfort and performance of the building. This paper proposes some recommendations for improving thermal comfort for similar housing projects in Alexandria, including: putting more focus on the passive design for cooling than heating; allowing wind flow for maximized natural ventilation cooling, using chimneys and other similar elements; using ventilated pitched roof spaces which allow to cool the house; using sun shading elements such as pergolas and projected porches in the proper facades and angles, to take advantage of the sun protection elements as well as the architectural style aesthetics, preferably moveable and interchangeable shading elements to function in both summer and winter.

## Data availability

### Underlying data

Data supporting reported results are available online at https://doi.org/10.5281/zenodo.4814710. ^23^

Data are available under the terms of the Creative Commons Attribution 4.0 International license (CC-BY 4.0).
