# Supplementary material for: Thermal comfort optimization through bioclimatic design in Mediterranean cities
Source: F1000Res. 2022 Jun 1;10:1047. Originally published 2021 Oct 15. [Version 2] doi: 10.12688/f1000research.73017.2 (PMC10616657; doi:10.12688/f1000research.73017.2)
Supplement: Peer review history

# Reviewer report: Reviewer response for version 2

**DOI:** 10.5256/f1000research.134544.r214715

**Published:** 2023-11-15

**Authors:** Klodjan Xhexhi

**Affiliations:** 1 Polis University, Tiranë, Albania

Regardless, please find below some recommendations.

  

1. Title: Thermal comfort optimization through bioclimatic design in Mediterranean cities.
  Recommendation: Thermal comfort optimization through bioclimatic design in Mediterranean cities . Case study Alex West, Alexandria, Egypt. According to my opinion, since the authors are dealing with a specific case study, it should be added to the title of the article.
2. The methodology and literature review are appropriate.
3. Section *“2.4 Thermal comfort design variables”*
  Recommendation: It is necessary to elaborate more on Fanger’s concept. Please introduce the inputs for energy balance such as metabolic rate, clothing insulation, air temperature, air velocity, mean radiant temperature, and relative humidity.
4. Energy usage is a significant factor that plays a crucial role in the bioclimatic architecture.
  Recommendation. Some specific data should be added especially in Alexandria’s case study. Heating and cooling loads are important to be mentioned.  
5. The quality of some of the drawings is not at the right level. Improvement should be made in Figure 5 and Figure 10. The position of the North is important to be present in the plans, in such a way that the readers can easily be oriented (Figure 5; 8; 10; 12).
6. Indoor thermal comfort is strictly related to the U-value coefficient (thermal performance coefficient), considering building materials.
  Recommendation:  Some specific data about the building envelope such as roof, terraces, walls, and windows should be considered in the section “ *Software project file settings and construction materials”.*
7. Specific climate data of the case study is important to be added such as wind speed and direction (wind rose), summer and winter temperatures, relative humidity, sunny days (degree days), and sun angle at least during the solstices.
8. The ventilation schemes due to simulations are appropriate.
  Recommendation: It is necessary to elaborate a little bit more related to the air temperature reduction (some specific data) due to cross and stack ventilation (Figure 19; 20), and air pressure disbalance.
9. Section *“Software used”*
  Recommendation: Elaborate a little bit more on the process of simulation using DesignBuilder per each scenario. It's not really clear.

However, the general approach of the paper, in my opinion, is quite nice and intriguing.

Is the work clearly and accurately presented and does it cite the current literature?

Yes

If applicable, is the statistical analysis and its interpretation appropriate?

Yes

Are all the source data underlying the results available to ensure full reproducibility?

Yes

Is the study design appropriate and is the work technically sound?

Yes

Are the conclusions drawn adequately supported by the results?

Yes

Are sufficient details of methods and analysis provided to allow replication by others?

Partly

Reviewer Expertise:

Bioclimatic Design, Building Materials, Technology of Architecture, Building Energy Efficiency

I confirm that I have read this submission and believe that I have an appropriate level of expertise to confirm that it is of an acceptable scientific standard.

# Reviewer report: Reviewer response for version 2

**DOI:** 10.5256/f1000research.134544.r214706

**Published:** 2023-10-30

**Authors:** Leila Sriti, Ahmed Kaihoul

**Affiliations:** 1 Department of Architecture, Mohamed Khider University, Biskra, Algeria; 2 Architecture, Mohamed Khider University of Biskra, Biskra, Biskra Province, Algeria

The paper, " *Thermal comfort optimization through bioclimatic design in Mediterranean cities*," investigates the impact of local Mediterranean bioclimatic strategies on thermal comfort in housing while examining architectural elements and treatments commonly associated with the so called “Mediterranean architecture” by the authors. The study comprises multiple sections, including the Introduction, Methods, Examples of Good Building Practices, Results, Discussion, and Conclusion.

**1.    Introduction:**

- The introduction provides a good context for the study, emphasizing the importance of bioclimatic design in different regions.
- The research aims are clearly stated.

Recommendation: The introduction could be enhanced with a more specific and concise problem statement to give readers a clear understanding of the study’s focus.

**2.    Methods:**

- The methods section is comprehensive and provides an excellent overview of the research approach.
- Previous studies are referenced to establish a foundation for the research.

Recommendation: Although the section is well-structured, we recommend to expand it with by including a summary of the research methodology; it may also be useful to provide a brief overview of the data collection process.

**3.    Examples of Good Building Practices:**

- This section presents three examples of passive and bioclimatic design in three Mediterranean cities. The selection criteria for these examples are explained.

Recommendation: The section could be improved with more detailed descriptions of the three examples, focusing on the architectural and bioclimatic elements that make them successful. Architectural drawings, diagrams and graphics could be used to illustrate the strategies applied in the analyzed examples. 

 Please correct the title: 3.4 Examples of mediterranean muilding features.

 We suggest removing the title 3.5 Results; and using the paragraph “ The paper investigates the impact of local Mediterranean building features … without the tested building features.” to introduce the next section (4. Analysis approach).

**4.    Analysis approach and 5. Case study building description**

- These two sections provide a detailed analysis of the case study, offering insights into the impact of different building features on thermal comfort.
- The data are presented effectively through tables and figures.

Recommendation: The section could benefit from a more concise presentation of the results before delving into scenario-specific details.

 We suggest changing the title numeration “5”. Case study building description” to “4.1.” Case study building description.

**6. Discussion:**

- The discussion section effectively interprets the results and compares different scenarios.
- The identification of the best bioclimatic design scenario is well-reasoned.

Recommendation: The discussion could be further enriched by linking the findings back to the broader context of bioclimatic design principles and architectural considerations.

**Conclusion and Recommendations:**

- The conclusion provides a clear summary of the study's findings, highlighting the significance of bioclimatic design and common Mediterranean building features.
- The recommendations are actionable and well-aligned with the research.

Recommendation: The conclusion section could include a concise summary of the main research contributions.

**Overall Assessment:**

- The paper offers valuable insights into the impact of buildings’ features on thermal comfort considering the Mediterranean climate.
- It successfully combines theoretical concepts with practical case study analysis.
- The language and organization are generally clear and effective, making it accessible to a broad audience.
- The study's conclusions and recommendations are well-supported by the research findings.
- The paper appropriately highlights the importance of passive cooling design, natural ventilation, and sun shading elements.

**Unassessed Aspects:**

- The paper's use of specific software tools and their settings were not evaluated due to their technical nature
  .
- It wasn’t possible to assess the accuracy of the numerical simulation results presented in the paper.

**Overall Recommendation:**

- The paper is well-structured and provides valuable information for architects and designers working in Mediterranean climate context. Enhancements in the introduction and conclusion sections and more detailed descriptions of the case study examples could further improve the paper.

Is the work clearly and accurately presented and does it cite the current literature?

Partly

If applicable, is the statistical analysis and its interpretation appropriate?

Not applicable

Are all the source data underlying the results available to ensure full reproducibility?

Partly

Is the study design appropriate and is the work technically sound?

Yes

Are the conclusions drawn adequately supported by the results?

Yes

Are sufficient details of methods and analysis provided to allow replication by others?

Partly

Reviewer Expertise:

Sustainable Build Environment; Building Physics Simulation; Thermal Comfort;  Vernacular Architecture

We confirm that we have read this submission and believe that we have an appropriate level of expertise to confirm that it is of an acceptable scientific standard, however we have significant reservations, as outlined above.

## References

[1] : The effect of climate-responsive design on thermal and energy performance: A simulation based study in the hot-dry Algerian South region. *Journal of Building Engineering* .2021;43: 10.1016/j.jobe.2021.103023 10.1016/j.jobe.2021.103023

# Reviewer report: Reviewer response for version 2

**DOI:** 10.5256/f1000research.134544.r139550

**Published:** 2022-08-12

**Authors:** Hassan Abdel-Salam

**Affiliations:** 1 Faculty of Engineering, Alexandria University, Alexandria, Egypt

This is a revised version submitted for publication. A first review of the manuscript was carried out resulting in some remarks that were adequately considered by the authors.

- This revised version has some modifications and constructive additions.
- Amendments were introduced to help in making the contents and statements easier to grasp by the readers.
- The closing part has been improved, and a language revision throughout the text.
- The manuscript in its current status stands as satisfactory, no further checks by this reviewer are required.

Is the work clearly and accurately presented and does it cite the current literature?

Yes

If applicable, is the statistical analysis and its interpretation appropriate?

Yes

Are all the source data underlying the results available to ensure full reproducibility?

Yes

Is the study design appropriate and is the work technically sound?

Yes

Are the conclusions drawn adequately supported by the results?

Yes

Are sufficient details of methods and analysis provided to allow replication by others?

Partly

Reviewer Expertise:

Architecture and Environmental Design

I confirm that I have read this submission and believe that I have an appropriate level of expertise to confirm that it is of an acceptable scientific standard.

# Reviewer report: Reviewer response for version 1

**DOI:** 10.5256/f1000research.76635.r97059

**Published:** 2021-12-01

**Authors:** Amin Habibi

**Affiliations:** 1 Department of Architecture, Faculty of Arts and Architecture, Shiraz University, Shiraz, Iran

I have some comments regarding this manuscript as follows:

1. A language revision is recommended.
2. The authors are strongly recommended to address more accurate references in the literature review to satisfy the previous works and establish the method. For example, see Ozarisoy & Altan (2021) for the latest review study on this area.
3. The data used as input in Designbuilder are not fully described.
4. There are no valid data regarding the simulation and it would be a lack of methodology, meanwhile, you do not validate your data by dataloggers it could not be reasonable. 
5. The conclusion should be further developed, a simple repetition of results should be avoided.

Is the work clearly and accurately presented and does it cite the current literature?

Partly

If applicable, is the statistical analysis and its interpretation appropriate?

I cannot comment. A qualified statistician is required.

Are all the source data underlying the results available to ensure full reproducibility?

Partly

Is the study design appropriate and is the work technically sound?

Partly

Are the conclusions drawn adequately supported by the results?

Partly

Are sufficient details of methods and analysis provided to allow replication by others?

Partly

Reviewer Expertise:

-

I confirm that I have read this submission and believe that I have an appropriate level of expertise to confirm that it is of an acceptable scientific standard, however I have significant reservations, as outlined above.

## References

[1] : Systematic literature review of bioclimatic design elements: Theories, methodologies and cases in the South-eastern Mediterranean climate. *Energy and Buildings* .2021;250: 10.1016/j.enbuild.2021.111281 10.1016/j.enbuild.2021.111281

# Response

**Published:** 2022-05-27

**Authors:** Nermine Hany

**Affiliations:** Arab Academy for Science, Technology & Maritime Transport, Egypt

Thank you for your important review. The authors worked on the points mentioned from your side and uploaded the updated version on the journal website.

 1. The authors are strongly recommended to address more accurate references in the literature review to satisfy the previous works and establish the method. For example, see Ozarisoy & Altan (2021) for the latest review study on this area.

- **The authors are grateful for the advice to refer to this reference. We referred to the reference and added more specific parts in the literature review to address Previous Studies on Modern Mediterranean ( in Methods Section)**

2. The data used as input in Designbuilder are not fully described.

- **The authors included a brief description of the case study project file settings assigned in DesignBuilder as the focus and the scale of the research was to give a generalized idea of the impact of local Mediterranean building features on the thermal comfort.**
- **All the Data supporting reported results are available online at https://doi.org/10.5281/zenodo.4814710.**

3. There are no valid data regarding the simulation and it would be a lack of methodology, meanwhile, you do not validate your data by dataloggers it could not be reasonable. 

- **Concerning data loggers, the authors believes that this is out of research scope due to unavailability of dataloggers and on-site field equipment.**

4. The conclusion should be further developed, a simple repetition of results should be avoided.

- **The authors updated the conclusion according to the first reviewer comments.**
- **The conclusion included a quick compacted summary of results, to be easier for quick reading.**

# Reviewer report: Reviewer response for version 1

**DOI:** 10.5256/f1000research.76635.r99740

**Published:** 2021-12-01

**Authors:** Hassan Abdel-Salam

**Affiliations:** 1 Faculty of Engineering, Alexandria University, Alexandria, Egypt

Developing strategies for reducing the overall energy consumption of buildings constitutes a core concern in the field of Architecture. Minimizing negative effects on the environment, while maintaining the desired indoor qualities / favorable conditions (such as thermal comfort) is a subject worthy of investigation.

 The study purpose and objectives are clear and targeted at enhancing the environmental performance of residential buildings, promoting the thermal comfort in indoor spaces, in addition to achieving optimization of economics in the built environment.

 The hybrid methodology adopted in the experiment is clear and helps establish then assess qualitative attributes based on clear/simple action-taking, measurement and inference of results.

 The paper has introductory theoretical reviews on the Bioclimatic Approach and the Mediterranean climatic context. The linkage of the two topics is used as a basis for adopting an environmentally driven thinking process.

 The section in pp. 5-10 has an analysis of three selected examples of good practice regarding passive and bioclimatic design in Mediterranean cities. This is followed by a case study of one relevant example in Alexandria, Egypt.

 Based on the review of the manuscript and its contents, the following issues are revealed:

- The paper could benefit from an overall re-arrangement under specific numbered headings or bulleted points in order to better present the research discourse and to follow the build-up of argument leading to findings and recommendations deduced from this study.
- The Conclusion Section on p.18 could also benefit from minor re-structuring under specific bulleted points in order to better present the research findings and to provide clearer guidance and / or specific recommendations addressed to designers.
  (Note: it would be useful if the author(s) can add concise statements about the validity of results and possibility of generalizing upon the findings across the broader Mediterranean zone).
- The text needs careful and thorough revision, and editing of language.

Is the work clearly and accurately presented and does it cite the current literature?

Yes

If applicable, is the statistical analysis and its interpretation appropriate?

Yes

Are all the source data underlying the results available to ensure full reproducibility?

Yes

Is the study design appropriate and is the work technically sound?

Yes

Are the conclusions drawn adequately supported by the results?

Yes

Are sufficient details of methods and analysis provided to allow replication by others?

Partly

Reviewer Expertise:

Architecture and Environmental Design

I confirm that I have read this submission and believe that I have an appropriate level of expertise to confirm that it is of an acceptable scientific standard, however I have significant reservations, as outlined above.

# Response

**Published:** 2022-05-27

**Authors:** Nermine Hany

**Affiliations:** Arab Academy for Science, Technology & Maritime Transport, Egypt

Thank you for your very informative and important review. The authors worked on the points mentioned from your side and uploaded the updated version on the journal website.

 1. The paper could benefit from an overall re-arrangement under specific numbered headings or bulleted points in order to better present the research discourse and to follow the build-up of argument leading to findings and recommendations deduced from this study.

- **The authors updated the headings arrangement and added more detailed headings (in red)**

2. The Conclusion Section on p.18 could also benefit from minor re-structuring under specific bulleted points in order to better present the research findings and to provide clearer guidance and / or specific recommendations addressed to designers.

- **The authors restructured the findings under 2 major bullet points and presented the recommendations in a more specific form. (In red)**

3. It would be useful if the author(s) can add concise statements about the validity of results and possibility of generalizing upon the findings across the broader Mediterranean zone.

- **The authors discussed the possibility of generalizing the findings at the conclusion and recommendations section.**

4.The text needs careful and thorough revision, and editing of language.

- **The authors generated a careful revision for language.**
